# Tracking Efficiency Improvement According to Incident Beam Size in QPD-Based PAT System for Common Path-Based Full-Duplex FSO Terminals

**DOI:** 10.3390/s22207770

**Published:** 2022-10-13

**Authors:** Siwoong Park, Chan Il Yeo, Young Soon Heo, Ji Hyoung Ryu, Hyun Seo Kang, Dong-Seon Lee, Jae-Hyung Jang

**Affiliations:** 1 School of Electrical Engineering and Computer Science, Gwangju Institute of Science and Technology, 123 Cheomdangwagi-ro, Buk-gu, Gwangju 61005, Korea; 2 Honam Research Center, Electronics and Telecommunications of Research Institute, 176-11 Cheomdan, Gwagi-ro, Buk-gu, Gwangju 61012, Korea; 3 Department of Energy Engineering, KENTECH Institute of Energy Materials and Devices, Korea Institute of Energy Technology, Naju 58330, Korea

**Keywords:** free space optical communications, optical wireless communications, quadrant photodiode, optical sensor, position measurement, tracking

## Abstract

Free space optical (FSO) communication can support various unmanned aerial vehicles’ (UAVs) applications that require large capacity data transmission. In order to perform FSO communication between two terminals, it is essential to employ a pointing, acquisition, and tracking (PAT) system with an efficient and optimal performance. We report on the development of a common optical-path-based FSO communication system, tailored for applications in UAVs. The proposed system is equipped with a quadrant photodiode (QPD)-based PAT system without an additional beacon beam subsystem. The presented approach reduced the structural complexity and improved the tracking efficiency for the same size, weight, and power (SWaP). To achieve a robust FSO link in a dynamic UAV environment, the observability and controllability were obtained based on the linearized control according to the incident beam size on the QPD, which was verified by optical simulation and experiments. As a result, the QPD-based PAT system for implementing FSO links demonstrated an up to 4.25 times faster tracking performance. Moreover, the FSO link experimentally confirmed the 1.25 Gbps full-duplex error-free communication at a 50 m distance.

## 1. Introduction

Various applications using unmanned aerial vehicles (UAVs) increasingly require large capacity and high-speed data transmission for inter-UAVs or between UAVs and ground control stations (GCS). The use of the millimeter wave band by fifth-generation mobile communication have increased, and the rapid depletion of the existing radio frequency spectrum have intensified. This phenomenon drives the development of ultra-broadband free space optical (FSO) communication, FSO-based application services, and an FSO-based network [1,2,3,4,5,6,7,8,9,10,11,12,13,14]. FSO communication features several advantages including no spectrum licensing, low-power consumption, electromagnetic interference immunity, enhanced channel security, and long-distance high-speed transmission [1,2,3,4,5,6,7]. Because FSO communication requires a line of sight (LOS) between FSO terminals, the pointing, acquisition, and tracking (PAT) system is essential for FSO communication [15]. To maintain uninterrupted LOS, various methods were proposed using a quadrant photodiode (QPD), modulated retro-reflector (MRR), fast steering mirror (FSM), and hybrid of QPD and FSM [15,16,17,18,19,20,21,22,23,24,25,26,27,28,29,30,31,32,33,34,35,36,37,38,39]. MRR-based PAT systems, represented by retroreflector free space optics, were mounted on UAVs for 560 m FSO communication, but they exhibited a limited performance in terms of transmission speed (Mbps) and simplex communication [16,17,18]. UAVs carrying a reflector only without TRx are lightweight but they cannot perform full-duplex communications. Full-duplex communication, which is essential for FSO network scalability and Gbps communication diversification, requires TRx’s on both the FSO terminals [9,10,11,12,13,14]. The FSM-based PAT system increases the control complexity and requires additional integrated modules for precision control [19,20,21]. Hybrid methods exhibit a good and stable performance. Nonetheless they present disadvantages, such as increased structural complexity, production costs, increased size and weight, and the need for an additional beacon beam subsystem with additional light sources [22,23,24,25,26,27,28]. The realization of a precise PAT system, which enables Gbps full-duplex transmission and maintains LOS, is more challenging in dynamic UAV environments. The high resolution and fast response speed of the QPD-based PAT system emerged as an interesting solution [29,30,31,32,33,34,35,36,37,38,39]. In addition, based on the incident beam size and incident beam position on the QPD, tracking efficiency was increased without additional components (i.e., FSM, deformable mirror, etc.).

Previously reported FSO communication systems employing an adaptive beamforming function [40] demonstrated an improved tracking efficiency between FSO terminals within a limited size, weight, and power (SWaP) value for UAV mounting.

In this study, a QPD-based PAT system without an additional beacon beam subsystem is proposed for FSO communications based on a common optical path. Furthermore, observability and controllability were confirmed by obtaining linearized control points from the QPD output according to the incident beam size on the QPD. Moreover, PAT was performed by controlling the pan and tilt unit (PTU). Therefore, precise LOS maintenance without additional beacon light sources and full-duplex Gbps high-rate data communication were simultaneously performed via a common optical path. We simulated and experimentally analyzed the QPD output and tracking efficiency depending on the incident beam size on QPD, based on a compact FSO PAT system.

## 2. Experimental Details

### 2.1. System Concept

**Figure 1:**
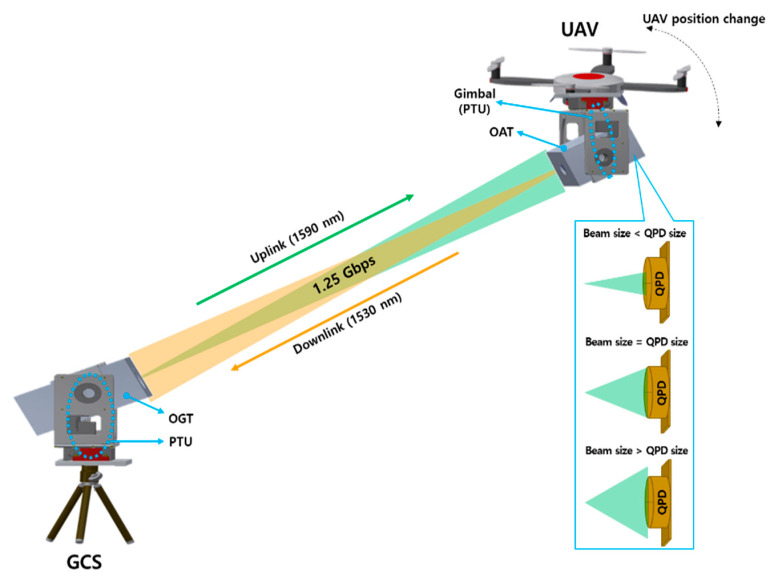
System concept of free space optical (FSO) communication between ground control station (GCS) and unmanned aerial vehicle (UAV).

Figure 1 illustrates the concept of the proposed FSO communication system. It is composed of a common path-based FSO terminal, which performs Gbps FSO communication between the GCS-UAV and the PTU/gimbal that controls horizontal (i.e., pan) and vertical (i.e., tilt) axes. This is essential for a PAT system to implement a robust FSO link. Here, the FSO terminal fitted to the GCS is referred to as an optical ground terminal (OGT), whereas that mounted on the UAV as an optical aerial terminal (OAT). The latter requires more detailed consideration due to its sensitivity to problems such as batteries, weight, center of gravity, vibration, and wind, etc. To reduce the detrimental effects of these issues, the OAT must be small and light-weight. The FSO terminal for Gbps full-duplex communication is more complicated compared to that for simplex communication because both terminals in OAT and OGT have TRx. Nevertheless, an FSO terminal supporting full-duplex communication is preferred over simplex communication due to the ease of configuring the FSO network between multiple UAVs and GCSs [9,10,11,12,13,14]. In addition, a PAT system for maintaining a robust LOS between the FSO terminals was also considered. It was made adaptable to changes in the datalink distance and reception beam size caused by thee dynamic environment of the UAV.

### 2.2. System Design

**Figure 2:**
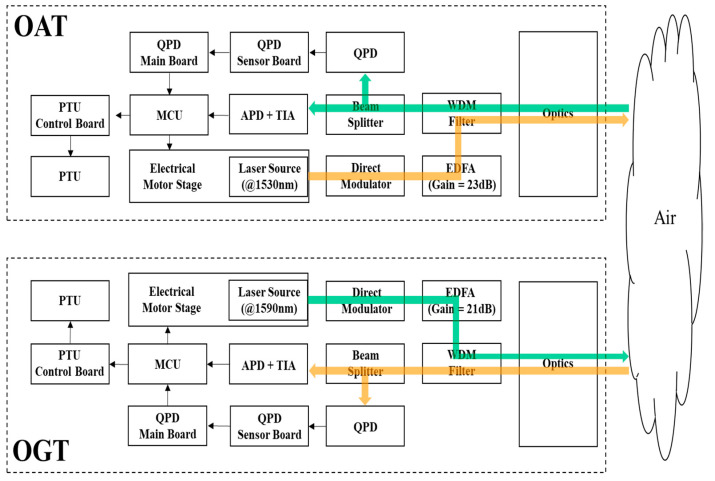
FSO communication system between OGT and OAT.

Figure 2 shows the overall structure of the proposed FSO system based on a common optical path. Each FSO terminal transmits and receives laser beams simultaneously in the common optical path. Data transmission and beacon functions were incorporated together to increase efficiency for SWaP without additional optical beacon subsystems. The InGaAs-based distributed feedback laser was selected as a light source for OGT and OAT, at wavelengths of 1590 nm (L-band) and 1530 nm (C-band), respectively. This was implemented to minimize possible interferences in the common optical path. If a common wavelength is shared in the TRx terminals, signal loss of at least 75% is expected due to the inevitably required 1:1 beam splitter (BS). The direct modulation method was utilized for non-return-to-zero on–off keying intensity modulation. To obtain sufficient optical gain, a L-band erbium-doped fiber amplifier (EDFA) with 21 dB gain and C-band EDFA with 23 dB gain were included in the OGT and OAT, respectively. To avoid unwanted light, anti-reflection coated band-pass filters (BF) were adapted in the first lens of optics inside the OGT and OAT. The customized plate type wavelength division multiplexing (WDM) filter prevents unwanted light from shining onto the avalanche photodiode (APD) and QPD. It is based on special coatings that distinguish transmitting and receiving beams. The WDM filter in the OAT reflects/transmits 1530/1590 nm light, and the WDM filter in OGT reflects/transmits 1590/1530 nm light. WDM filter also helps the received beam to safely enter the APD and QPD via a 7:3 BS. An additional BF is placed in front of the APD and QPD to remove the background noise. APD is employed for highly sensitive optical signal detection, and QPD provides precise location information to the PAT system. The output current of the APD was fed to a transimpedance amplifier (TIA) for low noise signal amplification. The microcontroller unit (MCU) controls the PTU and the electrical motor stage (EMS) by using the data produced by the QPD and the APD. The EMS is used to control the divergence angle of the transmission beam by controlling the light source position in the FSO terminal [40]. When the light source position is 0 mm in EMS, the divergence angle is the largest. As the location of the light source moves from 0 to 10 mm, the divergence angle decreases. The EMS can increase the scanning efficiency by increasing the detection probability for the target FSO terminal by initially adopting a large divergence angle [40]. Because the large divergence angle faces an extra power loss problem [40,41], an adaptive beam narrowing strategy was employed in the PAT system to compensate for extra power loss and to improve link robustness. The PAT system was operated in two modes. First, coarse tracking was carried out for scanning counter FSO terminals by adopting a large divergence angle. When the QPD in each FSO terminal began to produce the output signal, the PAT system started fine tracking. When the output of the QPD exceeded the threshold value, the LOS link was established and APD-based data communication starts. After the LOS link was established, an adaptive beam narrowing strategy could prevent link performance degradation in long distance datalink. The maximum angular velocity of the PTU was 1.0472 rad/s, and the angular resolution of the PTU was equally set to 0.025° for pan and tilt.

**Figure 3:**
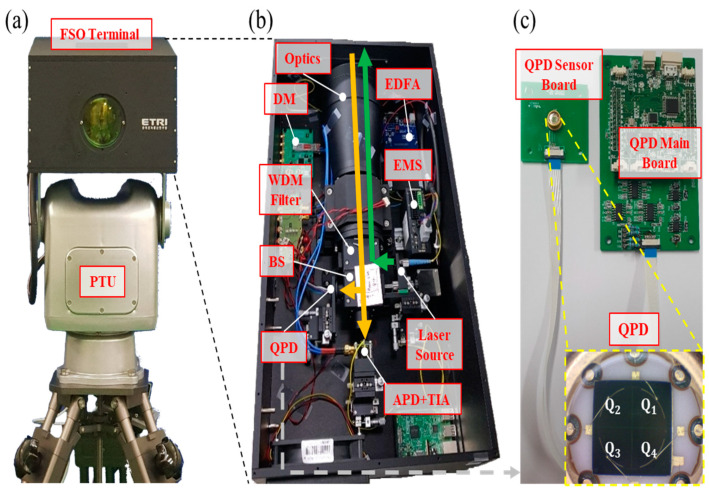
(**a**) FSO terminal on PTU, (**b**) inside view of FSO terminal, and (**c**) custom-designed quadrature photodiode (QPD) sensor board and main board for precision PAT.

Figure 3 shows the FSO terminal mounted on the PTU, its internal structure, custom-designed QPD main board, and QPD sensor board for precision PAT. The FSO terminals in the OAT and OGT are lighter than 8 and 12 kg, respectively. The OGT has a 100 mm aperture, and the total length of its optics is approximately 350 mm. The OAT has a 70 mm aperture, and the total length of its optics is approximately 250 mm. It is intentionally designed to be smaller than the OGT for practical mounting purposes onto UAVs. As can be seen from Figure 3b, APD and QPD are mounted on a three-axis stage having a 10 µm resolution for a precise optical alignment. The QPD with an active diameter of 2 mm consists of a 2 × 2 array of p-i-n photodiodes arranged counterclockwise in the *Q*_1_, *Q*_2_, *Q*_3_, and *Q*_4_ order, as shown in Figure 3c. The position information can be extracted by monitoring the signal from QPD by using the following equations [29,30,31,32,33,34,35]:

(1) $$V=V_{Q_{1}}+V_{Q_{2}}+V_{Q_{3}}+V_{Q_{4}}$$

(2) $$X\;=\;\frac{\left(V_{Q_{1}}+V_{Q_{4}}\right)-(V_{Q_{2}}+V_{Q_{3}})}{V},\;Y\;=\frac{\left(V_{Q_{1}}+V_{Q_{2}}\right)-(V_{Q_{3}}+V_{Q_{4}})}{V}$$

where $V_{Q_{1}}$, $V_{Q_{2}}$, $V_{Q_{3}}$, and $V_{Q_{4}}$ are the output voltage values of the four photodiodes *Q*_1_, *Q*_2_, *Q*_3_, and *Q*_4_, respectively. *V* is the total voltage produced by the four photodiodes. Equation (1) calculates the optical power incident to the QPD by summing up the signals detected by the photodiodes located in four quadrants. As the incident beam spot moves on the photosensitive surface of the QPD, the signal output of the four photodiodes in each quadrant changes according to the displacement of the beam spot. The (X, Y) indicating the position of the incident beam on the QPD can be calculated by using Equation (2). Consequently, pointing, acquisition, and tracking functions were performed by changing the pan and tilt of the PTU using the X and Y values obtained by the position of the incident beam on the QPD. In order to estimate the incident beam size on the QPD [36,37,38,39], the following parameters are defined.

(3) $$c_{h}=\frac{\Delta X}{\Delta \mathrm{PAN}}{\;,\;c}_{v}=\frac{\Delta Y}{\Delta \mathrm{TILT}}$$

Here, *c*_h_ is defined as the ratio of the change in the X value (ΔX) to the corresponding change in the pan angle (ΔPAN) moving on the horizontal axis. Similarly, *c*_v_ is defined as the ratio of the change in the Y value (ΔY) to the corresponding change in the tilt angle (ΔTILT) moving on the vertical axis.

(4) $$d_{h}=d_{\mathrm{QPD}}\times \frac{c_{h,\mathrm{ideal}}}{c_{h,\exp}}\;,\;d_{v}=d_{\mathrm{QPD}}\times \frac{c_{v,\mathrm{ideal}}}{c_{v,\exp}}$$

In Equation (4), *d*_h_ is the horizontal width of the incident beam on the QPD, *d*_QPD_ is the diameter of the QPD, and *d*_v_ is the vertical length of the incident beam on the QPD. The initially set *c*_h, ideal_ and *c*_v, ideal_ are the ideal values when OAT and OGT are well aligned and the diameter of the incident beam matches the QPD diameter. The *c*_h, exp_ and *c*_h, exp_ are the experimental values. Using Equations (3) and (4), the incident beam size on the QPD is estimated by comparing the ideal and experimental values.

## 3. Experimental Results

To reduce the impact of atmospheric conditions on the datalink performance, this experiment was conducted in a stable and clear atmospheric condition at a 50 m datalink distance. In addition, to increase the margin of QPD-based tracking, the beam reaching the counterpart FSO terminal should be as large as possible. The position of the light source on the EMS at each FSO terminal was initially set to 0 mm, making the beam size maximum at the counterpart FSO terminal.

**Figure 4:**
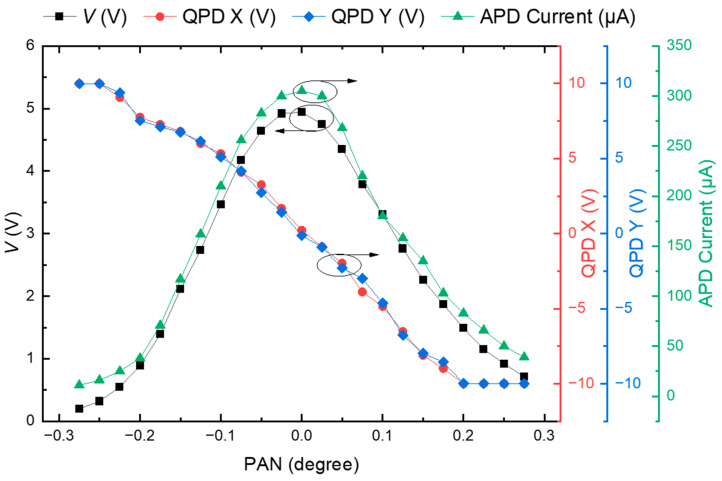
Result of *I* value of QPD, X value of QPD, Y value of QPD, and current value of APD with pan angle variation of PTU (incident beam size on the QPD = QPD size).

Figure 4 shows the variation of *V*, X, and Y of the QPD, and the current value of APD depending on the pan angle, when the incident beam size matches the size of the QPD. When the X value of the QPD approaches zero, the *V* value of the QPD and the output current (*I*) of the APD are also maximized. Due to the 2D symmetry of the Gaussian beam incident to the QPD, the output of QPD changes identically for the same change in the pan and tilt angles. Therefore, experiments were conducted only for the pan angle variations.

**Figure 5:**
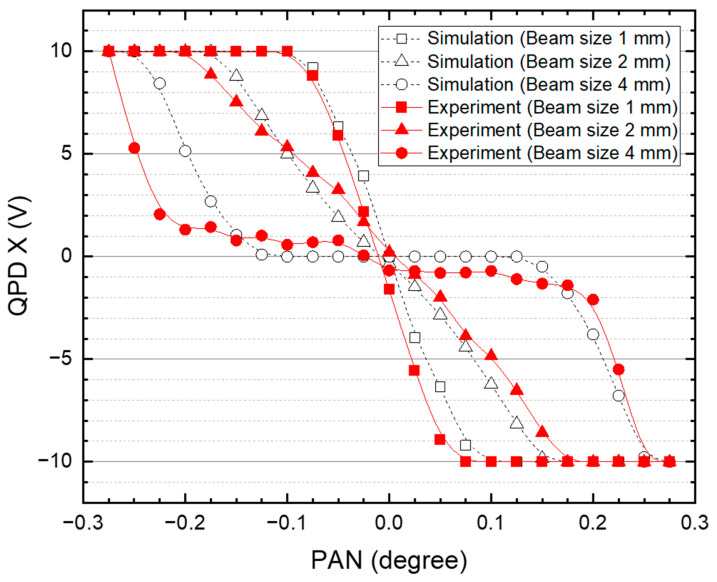
Result of X value of QPD based on incident beam size on QPD with respect to the change of pan angle of PTU. The dotted and solid line represent the simulation and experimental results, respectively.

Figure 5 shows the output of the X value of the QPD with respect to the change in the pan angle of the PTU for various incident beam sizes on the QPD. Simulations and experiments were performed based on three conditions between the incident beam diameter on the QPD and the size of the QPD. The light source used in the simulation had an optical power of 200 mW and Gaussian beam profile. The following attributions were defined: “Beam size 1 mm” corresponds to the case when the incident beam size on the QPD is half the size of QPD; “Beam size 2 mm” to the case when both sizes are the same; “Beam size 4 mm” to the case when the incident beam size on the QPD is twice the size of QPD. To change the incident beam size on the QPD, the focus was adjusted via the *z*-axis of the three-axis manual stage of the QPD. The size of the incident beam was confirmed by scanning the output value of the QPD through the *x* and *y*-axes travel of the three-axis manual stage. When the incident beam size on the QPD was approximately equal to the size of the QPD, the angle control points of the PTU that control the incident beam on the QPD to the center of the QPD had a linear resolution. In addition, these results produced similar results for the Y value of QPD. When the incident beam size became smaller than the size of QPD, the span of the pan angle became narrower, reducing the dynamic range of the angle control of the PTU in tracking control. When the incident beam size was larger than the size of QPD, the flat dead zone was formed on the X value of QPD output, making it difficult to control the angle of the PTU, which was responsible for controlling the position of the incident beam to the center of the QPD. As shown in Figure 5, it was confirmed that the outputs of QPD according to the incident beam size in the simulation and experimental results are similar.

**Figure 6:**
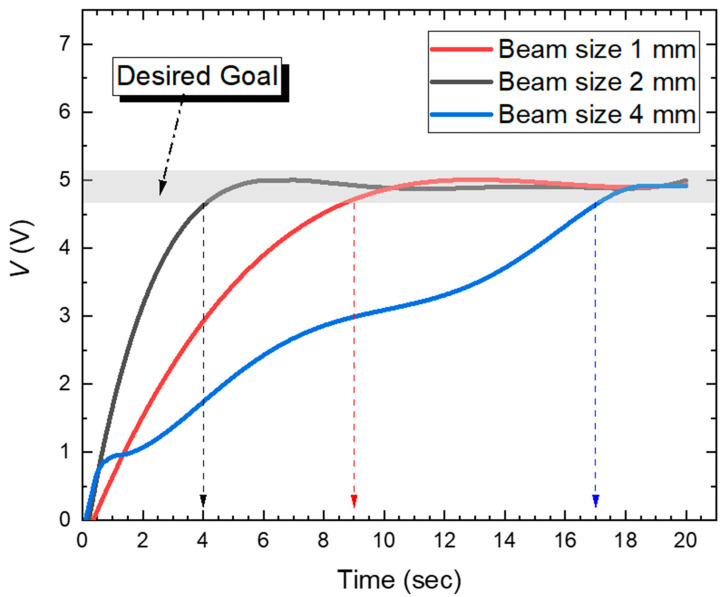
Tracking time depending on the incident beam size on QPD.

Figure 6 shows the tracking time depending on the incident beam size on the QPD obtained through the experiment. The case of “Beam size 2 mm” is the fastest to reach the desired goal compared to the other incident beam sizes on the QPD. On the other hand, when the beam sizes were smaller/larger as indicated by the red/blue curves, the measurement took 2.5/4.25 times longer. These results show that when “Beam size 1 mm” and “Beam size 2 mm” are based on Figure 5 and Figure 6, the QPD generates a linearized output for the received beam and provides linear resolution for the control points of the QPD, helping to reach the desired goal region during tracking control. Among them, in the case of “Beam size 2 mm”, the dynamic range (i.e., control point) is relatively wider than in the case of “Beam size 1 mm”, so it is possible to obtain a linearized output for all control positions. It is thus confirmed that the desired goal region can be reached fastest when a tracking algorithm is performed. This strongly indicates that the incident beam size on the QPD should be considered for the optimized QPD-based PAT system.

**Figure 7:**
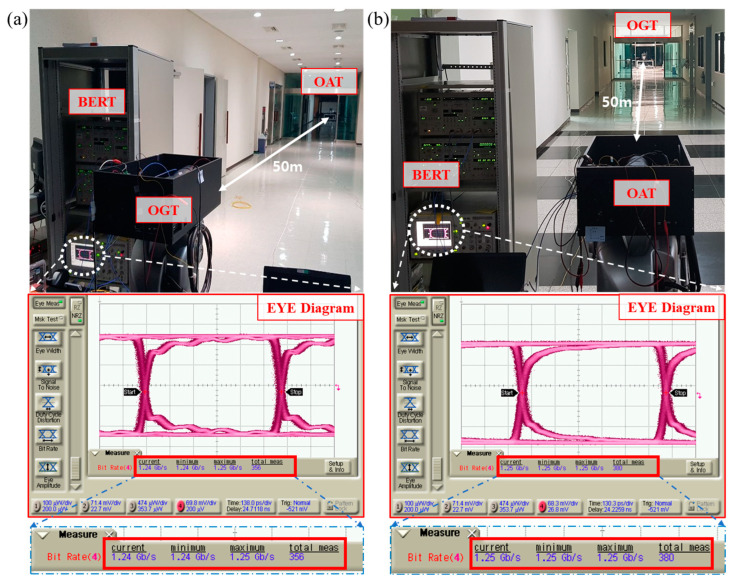
Bit error rate (BER) measurement of FSO link between OGT and OAT at 50 m distance: (**a**) OGT to OAT and (**b**) OAT to OGT.

Figure 7 shows the measured bit error rate (BER) of the FSO communication link between OGT and OAT at a 50 m distance. BER was measured with the non-return to zero (NRZ) format pseudorandom binary sequence (2^7^−1) data stream using pulsed pattern generator (Anritsu MP1763C) and error detector (Anritsu MP1764C). The measured laser output power at the output fiber and the sensitivity of the APD were approximately 200 mW and −32 dBm, respectively. The 1.25 Gbps error-free (BER < 10^−12^) data communication was verified with 1.25 Gbps at the 50 m distance.

Various PAT systems have been reported for mobile FSO communication as compared in Table 1. The MRR-based mobile FSO communication system employs TRx only on the ground because it uses simplex method. Accordingly, the platform mounted on the UAV has the advantages of a small size, lightweight, and simple structure [16,17,18], but the ground terminal carries the burdens caused by the separated optical communication paths and beacon beam tracking. In addition, it has limitations in the datalink distance and modulation speed. Recent studies have reported 500 Mbps at the distance of 560 m between UAVs and the ground [18]. The German aerospace center (DLR) performed 1.25 Gbps FSO communication between the airborne platform flying at a maximum speed of Mach 0.7 and the ground [24]. However, it required a large number of optical components due to the use of separate optical paths for data communication and beacon beam tracking, simplex methods, and the hybrid PAT system. The naval research laboratory (NRL) in the United States conducted full-duplex 1 Gbps FSO communication using a five-cell combinational detector capable of tracking and communication with one detector [25]. However, the control complexity and the number of optical components increased by using the hybrid PAT and separated optical path for transmitter and receiver [25]. The national institute of information and communication (NICT) in Japan performed the first statistical analysis on 100 m simplex communication channels between the ground and UAV equipped with corner-cube retro-reflector and camera [26]. Since transceivers share optical paths in this system, the number of the required optical components is smaller than that of MRR-based mobile FSO systems. Our mobile FSO system, sharing optical paths for data transmission and beacon beam tracking and using QPD-based PAT systems, performed 1.25 Gbps full-duplex communication with the least optical components compared to other studies. The performance was evaluated in a stable and clear atmospheric condition at 50 m datalink distance. The effect of the realistic atmospheric environment and vibration of unwanted UAVs on the performance of FSO links will be analyzed in the future to optimize the PAT system.

## 4. Conclusions

A FSO communication system employing a QPD-based PAT and a common optical path scheme is reported. The precise LOS maintenance and full-duplex 1.25 Gbps high-rate data communication were performed simultaneously at 50 m. The 4.25 times faster tracking performance was experimentally demonstrated when the incident beam size on the QPD became approximately equal to the size of the QPD. The improved tracking performance could help to quickly establish a reliable mobile FSO communication link. We believe that the proposed full-duplex Gbps FSO communication system with a common optical path and a simple and compact structure has the potential to be used for future FSO-based fronthaul/backhaul in 5G wireless networks and application services using UAV.

## Figures and Tables

**Table 1:** FSO communication system for UAVs with PAT system.

Type of Data Transmission	Optical Path for Tx/Rx/Beacon	Link Rate (Gbps)	Beamforming (*θ*_div_)	PAT Type	Number of Optical Components	Ref.
Simplex (UAV-Ground)	Separate	0.5	SLM ^1^	Hybrid (MRR + FSM + QPD + Camera)	≤19 (Ground)	[18]
Simplex (Airborne platform -TOGS)	Separate	1.25	Fixed (2.26 mrad)	Hybrid (FSM + Camera)	≥23 (TOGS ^2^)	[24]
Full-duplex (UAV-Ground)	Separate	1	FSM	Hybrid (FSM + 5-cell detector)	≤10 (Ground)	[25]
Simplex (UAV-Ground)	Share	-	-	Hybrid (FSM + Camera + QPD)	≤12 (Ground)	[26]
Full-duplex (OAT-OGT)	Share	1.25	EMS (0~8.84 mrad)	QPD	≤9 (OGT)	This work

^1^ SLM: spatial light modulation, ^2^ TOGS: transportable optical ground station.

## Data Availability

Not applicable.
